# A fluorescent approach for identifying P2X1 ligands

**DOI:** 10.1016/j.neuropharm.2015.05.016

**Published:** 2015-11

**Authors:** Marc-David Ruepp, James A. Brozik, Iwan J.P. de Esch, Richard W. Farndale, Ruth D. Murrell-Lagnado, Andrew J. Thompson

**Affiliations:** a Department of Pharmacology, Tennis Court Road, Cambridge CB2 1PD, UK; b Department of Chemistry and Biochemistry, University of Bern, Freiestrasse 3, CH-3012 Bern, Switzerland; c Medicinal Chemistry, VU University Amsterdam, Amsterdam, The Netherlands; d Department of Biochemistry, University of Cambridge, Cambridge CB2 1QW, UK; e Washington State University, Department of Chemistry, Pullman, WA 99164-4630, USA

**Keywords:** Fluorescent, Ligand binding, Ligand gated ion channel, P2X1, Fragment-based drug discovery, Pharmacology, NF449, Alexa-647, FBDD, Fragment-based drug design, ATP, Adenosine triphosphate, SCA, Scaffold classification approach

## Abstract

There are no commercially available, small, receptor-specific P2X1 ligands. There are several synthetic derivatives of the natural agonist ATP and some structurally-complex antagonists including compounds such as PPADS, NTP-ATP, suramin and its derivatives (e.g. NF279, NF449). NF449 is the most potent and selective ligand, but potencies of many others are not particularly high and they can also act at other P2X, P2Y and non-purinergic receptors. While there is clearly scope for further work on P2X1 receptor pharmacology, screening can be difficult owing to rapid receptor desensitisation. To reduce desensitisation substitutions can be made within the N-terminus of the P2X1 receptor, but these could also affect ligand properties. An alternative is the use of fluorescent voltage-sensitive dyes that respond to membrane potential changes resulting from channel opening. Here we utilised this approach in conjunction with fragment-based drug-discovery. Using a single concentration (300 μM) we identified 46 novel leads from a library of 1443 fragments (hit rate = 3.2%). These hits were independently validated by measuring concentration-dependence with the same voltage-sensitive dye, and by visualising the competition of hits with an Alexa-647-ATP fluorophore using confocal microscopy; confocal yielded *k*_on_ (1.142 × 10^6^ M^−1^ s^−1^) and *k*_off_ (0.136 s^−1^) for Alexa-647-ATP (*K*_d_ = 119 nM). The identified hit fragments had promising structural diversity. In summary, the measurement of functional responses using voltage-sensitive dyes was flexible and cost-effective because labelled competitors were not needed, effects were independent of a specific binding site, and both agonist and antagonist actions were probed in a single assay. The method is widely applicable and could be applied to all P2X family members, as well as other voltage-gated and ligand-gated ion channels.

This article is part of the Special Issue entitled ‘Fluorescent Tools in Neuropharmacology’.

## Highlights

• A novel fluorescence-based screening approach for identifying P2X1 receptor ligand candidates.
• Fragment-based drug discovery applied to ligand-gated ion channels.
• The use of confocal microscopy to determine the kinetics and affinity of Alexa-647-ATP binding to P2X1 receptors.
• Alexa-647-ATP for imaging P2X1 receptors on live cells.

## 1 Introduction

The P2X1 purinergic receptor, is a transmembrane ligand-gated ion channel that is distributed in cells of the blood, such as platelets and polymorphonuclear neutrophils, and in the smooth muscle of organs such as the urinary bladder, vas deferens, arteries and intestines (Gever et al., 2006, White et al., 2013, Darbousset et al., 2014). Their activation results in the opening of an integral cation channel that leads to membrane depolarisation and calcium entry.

Currently there is clinical potential for P2X1-selective ligands that has not been met (Gunosewoyo and Kassiou, 2010). For example, platelets maintain blood vessel integrity after injury and their activation is the first event in the process of haemostasis that prevents blood loss. In healthy individuals endothelium-derived inhibitors (prostacyclin and nitric oxide) prevent activation, but upon damage to blood vessels a local loss of these inhibitors facilitates platelet activation by molecules such as exposed collagen and diffusible secondary agonists such as ADP, thromboxane A_2_ and thrombin (Jackson, 2007). Activation intimately relies upon increased cytosolic Ca^2+^, and cell-surface P2X1 receptors are major contributors as they amplify the responses that cause blood clotting (Fung et al., 2007, Tolhurst et al., 2008, Varga-Szabo et al., 2009, Fung et al., 2012). All steps in platelet activation are potential targets for anti-thrombotics, but current treatments, such as Clopidogrel, Prasugrel, Ticlopidine and aspirin, can also cause unwanted bleeding. In contrast, P2X1 receptors are only important under high shear stress such as that found in narrow and stenotic vessels, the very places where blood clots are most dangerous. Consequently, P2X1 receptor inhibition has a lower bleeding risk because under normal arterial flow the receptors are unaffected and normal haemostasis is maintained. This lower risk makes P2X1 receptors a desirable target for preventing blood clots.

In addition to the antithrombotic potential of P2X1 receptor antagonists, there is also strong evidence that these inhibitors could be combined with α_1A_-adrenoreceptor antagonists to produce a highly efficient and fully reversible non-hormonal male contraceptive. Joint adrenergic and P2X1 receptor inhibition blocks sperm transport by preventing the smooth muscles around the vas deferens from contracting, causing reversible infertility without the undesirable sexual, behavioural, physiological, and psychological side effects that are seen with hormonal drugs (White et al., 2013). As there is already an existing and widespread use of α_1_-adrenoceptor antagonists for the treatment of disorders such as hypertension, panic, anxiety and benign prostatic hyperplasia, only the development of a suitable P2X1 receptor antagonist is required before the benefit of this pharmacological strategy can be realised.

One means to identify new drug candidates for the P2X1 receptor is fragment-based drug-discovery (FBDD), an efficient and rational approach that is becoming widely adopted by industry and academia (Murray et al., 2012, de Graaf et al., 2013, Joseph-McCarthy et al., 2014). The chemical space of molecules has been enumerated up to 17 atoms of C, N, O, Cl and S (termed heavy atoms), following simple rules of stability and synthetic feasibility, forming the GDB-17 database that contains 167 billion structures (Reymond et al., 2012). Because this number is so vast, industry typically screens smaller compound libraries (100,000 +) at a single concentration. However, even this library size is resource-intensive, does not always identify effective ligands and can be hindered by a legacy of previous targets (Macarron, 2006). It has therefore become recognised that screening smaller compounds (or fragments) can be more effective. Using FBDD, **1**/diverse chemical space can be efficiently described by smaller numbers of lower molecular weight and lower complexity compounds, **2**/lower complexity raises the chance of identifying protein-ligand interactions (Hann et al., 2001), **3**/small fragment libraries (∼1000 compounds) typically result in a higher hit rate than when screening drug-like compounds (Schuffenhauser et al., 2005, Albert et al., 2007, Siegal et al., 2007, Chen and Hubbard, 2009, Gupta et al., 2009), **4**/hit fragments are easier to optimize to improve their affinities and drug-like properties, and **5**/FBDD is design-intensive rather than resource-intensive (Rees et al., 2004). Using this method we have had previous success in identifying novel drugs for other ligand-gated ion channel targets (Thompson et al., 2012, Verheij et al., 2012, Thompson et al., 2013).

Here we describe the use of FBDD in the search for P2X1 receptor ligands. Owing to the need for medium-throughput, reduced material costs, and the difficulties that the very rapid desensitisation of P2X1 receptors can create for electrophysiological-based methods, a fluorescence-based approach was used.

## 2 Materials and methods

### 2.1 Materials

Alexa-647-ATP was from Life Technologies (CA, USA). Human P2X1 (Accession: P51575) subunit cDNA was provided by Richard Evans (Leicester University, UK).

### 2.2 Plasmids

A FLAG-tagged human P2X1 subunit expression construct was created by fusion PCR. The fragment was cloned together with an IRES-IFP1.4 (Shu et al., Science 2009; 324 no.5928) fusion PCR product as three point ligation into the MluI/SpeI digested pLV-T backbone (Marquis et al., 2009). The bicistronic mRNA expressed from the EF1alpha promoter codes for a c-terminal FLAG-tagged P2X1 protein and IFP1.4. The pLV-tTR-Krab-Blast vector was created by exchanging the IRES-dsRed cassette from pLV-tTR-Krab-dsRed with an IRES-Blasticidin expression cassette. All constructs were verified by DNA sequencing.

### 2.3 Cell lines

HEK 293T cells were first transduced with pLV-tTR-Krab-Blast to create the HEK293-tTR-Krab-Blast cell line. After selection of transduced cells with Blasticidin, this cell line was transduced with pLV-P2X1-IRES.IFP1.4 to create the inducible P2X1-FLAG-IRES-IFP1.4 cell line.

### 2.4 Virus production

FuGene HD (Promega, WI, USA) was used to transfect HEK 293T cells with pLV-tTR-Krab Blast and pLV-P2X1-FLAG-IRES-IFP1.4, respectively along with pCMVΔR8.91 and pMD2.G according to established methods (Wiznerowicz and Trono, 2003). Lentiviral supernatants were collected 48, 72 and 96 h post transfection and filtered through a 0.45 μM polyethersulfone sterile filter (Millipore Corp, MA, USA). For transduction, HEK 293T cells were incubated with lentiviral supernatants supplemented with 5 μg mL^−1^ polybrene (hexadimethrine bromide, Sigma Aldrich St. Louis, MO, USA). After 7 h, polybrene was diluted to 2.5 μg mL^−1^ by the addition of fresh DMEM/10% FBS, and the procedure was repeated for two days.

After expansion of the double transduced cells, doxycycline and biliverdine were added to the medium for 48 h and highly IFP1.4 positive cells were collected by fluorescence-activated cell sorting (FACS) to yield a highly transduced cell pool. High level expression was further optimized by generating a clonal cell line. This was achieved by visual inspection of Alexa-647-ATP binding using confocal microscopy.

### 2.5 Screening library

The screening compounds are commercially available via IOTA Pharmaceuticals (IOTA Pharmaceuticals Ltd, Cambridge, UK). This library was derived from a larger proprietary compound collection and the selection was loosely based on the rule-of-three criteria and aimed at maximal structural diversity (Congreve et al., 2003; de Graaf et al., 2013).

### 2.6 Plate preparation

For each screen two 96-well microplates plates were prepared. The first of these plates contained 80 different fragments in separate wells of columns 2–11, an ATP concentration–response (1 mM–3 nM in 3-fold dilutions) in column 1, and a saline control in column 12. In the second plate, columns 1–11 contained 30 μM ATP and column 12 was an ATP concentration–response (1 mM–30 nM in 3-fold dilutions).

### 2.7 Fluorometric microplate assays

Assays were performed using methods similar to those described elsewhere (Thompson et al., 2010). In brief, fragments were assessed for both agonist and antagonist activities using HEK 293T cells stably expressing doxycycline-inducible P2X1 receptors incubated for 45 min with 50 μl of a fluorescent dye sensitive to membrane potential (dye FLIPR Membrane Potential Assay Kit, Molecular Devices, Wokingham, UK) that was dissolved in buffer (mM: 115 NaCl, 1 KCl, 1 MgCl_2_, 1 CaCl_2_, 10 HEPES, 10 d-Glucose, pH 7.4). Dye-loaded cells were transferred to a FlexStation III (Molecular Devices) where compound applications were made without removing the dye. At recording intervals of 1.2 s, baseline fluorescence was recorded for 20 s, followed by a further 60 s after the addition of compounds. Ultimately two applications were made to the same cells. In the first, agonist activity was assessed by adding 50 μl novel fragment (final concentration = 300 μM) to each well. Following the completion of the first application, a second 50 μl application of 30 μM ATP (final concentration = 10 μM) was then added to the same cells. A response upon the first (fragment) application and an absence of a response on the second (ATP) application indicated agonist activity followed by inhibition as a result of receptor desensitisation. In contrast, an antagonist did not elicit a response in the first application and inhibited the subsequent ATP response. The absence of a response during the first application and a robust ATP response upon the second application indicated that the fragment was inactive.

In addition to testing fragments, the reproducibility of each experiment was assessed by including an ATP concentration–response in the first column and a saline control in the last column of the first application. This ensured ATP activation at appropriate concentrations at the start of the experiment and that buffer alone did not alter the baseline fluorescence. The saline control in the first application also meant that an ATP concentration–response could be performed at the end of the second application. This allowed cell viability to be assessed by comparing the ATP concentration–response at the start of the first application with the ATP concentration–response at the end of the second application. At least two replicates were performed and compounds scored as hits when activity was observed on two plates. Following the identification of active compounds from these single-concentration screens, the same procedure was used to subsequently test hits for concentration-dependence to confirm their activity and to obtain a measure of their potency. Measurements from these concentration-dependence experiments were fitted using Eq. (1) below.

Applying novel compounds and a known agonist to the same cells had several advantages, namely that 1) agonist, antagonist and allosteric modulators were identified in a single screen, 2) which reduced material costs, 3) the 16 min time period between the first and second applications allowed fragments to equilibrate in the presence of the cells before the second ATP application was added and, 4) reproducibility was improved because the same cells were subjected to both applications.

### 2.8 Data analysis

Measurements from the FlexStation III were recorded using the integrated software SoftMax Pro v5.4.5 (Molecular Devices). Analysis of concentration-dependence was performed with GraphPad Prism v5.00 (GraphPad Software, San Diego, CA, USA). Peak fluorescence was measured at a range of ligand concentrations and normalised to the maximum peak fluorescence for the same experiment. The mean and standard error of the mean (S.E.M.) for a series of measurements was plotted against agonist or antagonist concentration and iteratively fitted to the following equation:

(1) $$y=I_{\min}+\frac{F_{\max}-F_{\min}}{1+10^{\left({\left(\log R_{50}-x\right)}^{n_{H}}\right)}}$$

where *F*_min_ is the baseline fluorescence, *F*_max_ is the peak fluorescence, *R*_50_ is the concentration of ligand needed to generate a half-maximal response, *x* is the ligand concentration and *n*_*H*_ is the Hill slope.

### 2.9 Confocal microscopy

Live cells were routinely visualised in phosphate buffered saline (PBS (mM); 155 NaCl, 3.0 Na_2_HPO_4_, 1.0 KH_2_PO_4_, pH 7.4). Static and time-lapse images were collected using a FV300 laser-scanning confocal microscope with either an Olympus UplanFLN 40 × NA1.30 oil immersion objective and a 60 μm confocal aperture, or a PlanApo 60x NA1.42 oil immersion objective and a 60 μm confocal aperture. Images were exported to ImageJ v1.47 (National Institutes of Health, USA) and the Multi Measure plugin (Optinav Inc, WA, USA) used for analysing regions of interest.

### 2.10 Kinetic analysis

Kinetic parameters were determined according to the following model of a simple bimolecular binding scheme:

(2) $$L+R\overset{k_{\text{on}}}{\underset{k_{\text{off}}}{⇌}}LR$$

where *L* is the free ligand concentration, *R* is receptor concentration, *LR* is the ligand–receptor complex and *k*_on_ and *k*_off_ are the microscopic association and dissociation rate constants. In a simple scheme such as this, the equilibrium dissociation constant (*K*_d_) is equal to the ratio of dissociation to association rate constants, such that:

(3) $$K_{b}=\frac{k_{-1}}{k_{+1}}$$

According to a one site binding model of the type shown, the observed rates of association and dissociation can be used to estimate *k*_+1_ and *k*_−1_:

(4) $$1/\tau_{off}=k_{-1}$$

and

(5) $$1/\tau_{\text{on}}=k_{+1}\left[L\right]+k_{-1}$$

where τ_on_ refers to the rate at which fluorescence increases, τ_off_ refers to the rate that fluorescence decreases and [*L*] is concentration of the ligand used.

Association was determined by measuring the increase in fluorescence seen at the cell surface following the application of different concentrations of Alexa-647-ATP. Dissociation was measured by 3 rapid washes with PBS on Alexa-647-ATP equilibrated cells. Intracellular and background fluorescence was also routinely monitored as a control.

## 3 Results

### 3.1 Effects of a known agonist and antagonists

**Fig. 1:**
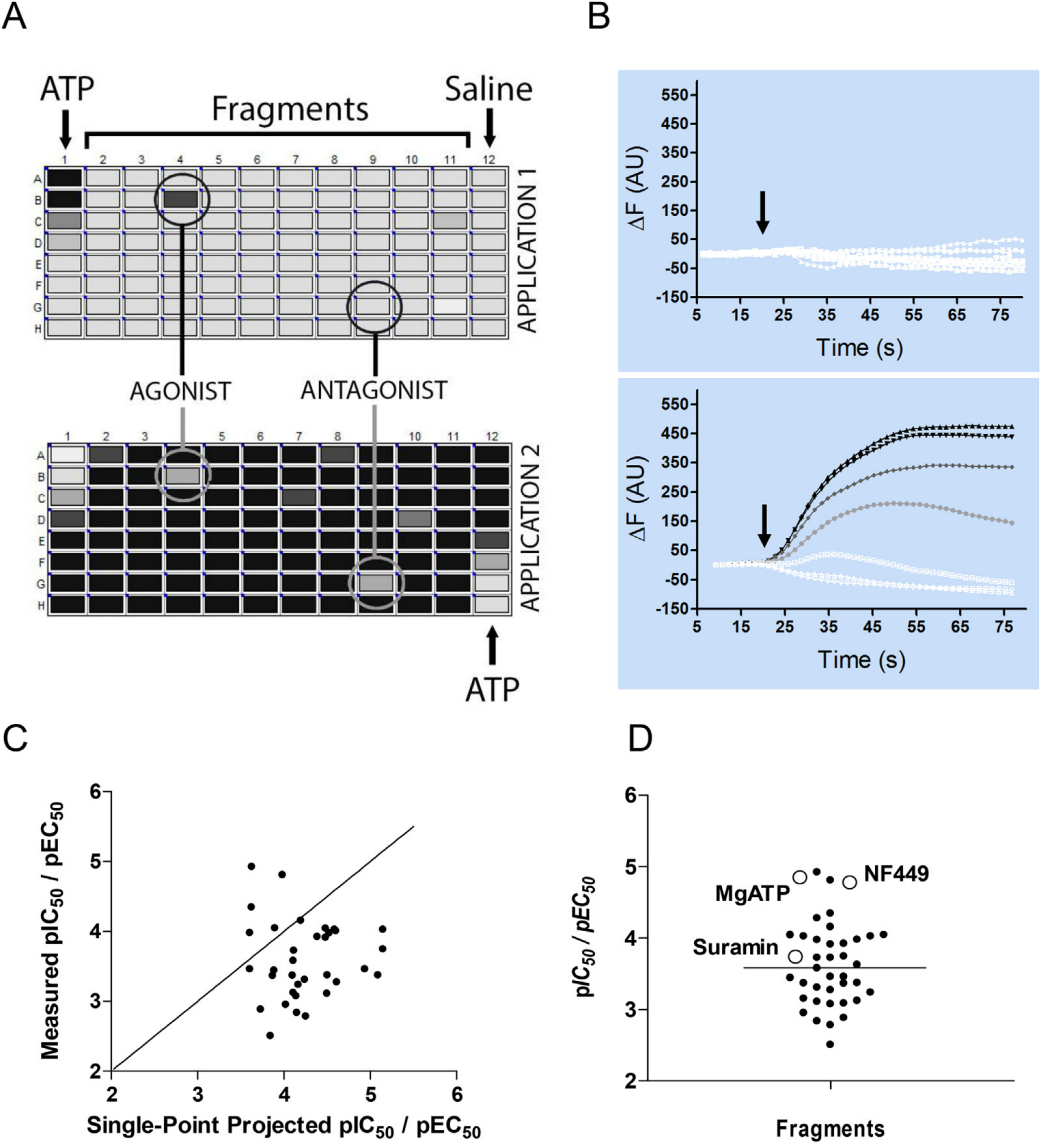
Typical data from fluorescent microplate assays using stably expressed doxycycline-inducible P2X1 receptors in a HEK 293T cell line. (A) An ATP concentration–response is included in column 1 of application 1, while column 12 contains saline alone. For hit screening 300 μM of different fragments are added to each well in columns 2–10. Because column 12 of application 1 contains saline alone, an ATP concentration–response can be performed on the same cells during application 2; ATP concentration–response curves at the start and end of the experiment can then be compared to confirm the consistency of the responses throughout the experimental period. To make visualisation of large datasets simpler, peak fluorescent responses are binned and assigned colours by the acquisition software, ranging from the largest fluorescence change (Black) to the smallest (white). Fragments are defined as agonists, antagonist, or inactive depending upon the responses to the two applications; see the methods section for a description. (B) The top panel shows a lack of fluorescent response following the addition of varying concentrations of ATP (arrow) to uninduced HEK 293T cells. The bottom panel shows raw data from an identical ATP application (arrow) on doxycycline-induced HEK 293T cells. The data in the bottom panel is from column 1 of Fig. 1A, plotted as a change in fluorescence over time. In both panels a baseline is recorded for 20 s before the addition of ATP. Such data can be used to plot concentration–dependence curves similar to those shown in Fig. 2. When hit fragments are identified at a single (300 μM) concentration, their concentration–dependence can be later assessed using the same experimental protocol as the ATP standards shown here. Although P2X1 receptors desensitise rapidly when measured using electrophysiological techniques, the fluorometric method shown here has much longer lasting responses as the cells are not voltage-clamped (depolarisation is longer lived); similar long-lived responses are seen for the rapidly desensitising 5-HT_3_ receptor when using the same dye (Price and Lummis, 2005). (C) A comparison of p*IC*_50_ values estimated from single-point measurements (300 μM) and those calculated from concentration-dependence curves. The straight line shows the vector along which the single-point predictions and measured potencies would be equal. (D) A comparison of p*IC*_50_ values determined for identified hit fragments (*closed circles*), ATP (*open circle*) and the established P2X1 antagonists, NF449 and suramin (*open circles*). Each point represents a different compound and the average affinity of all the fragments is shown as a horizontal bar.

**Fig. 2:**
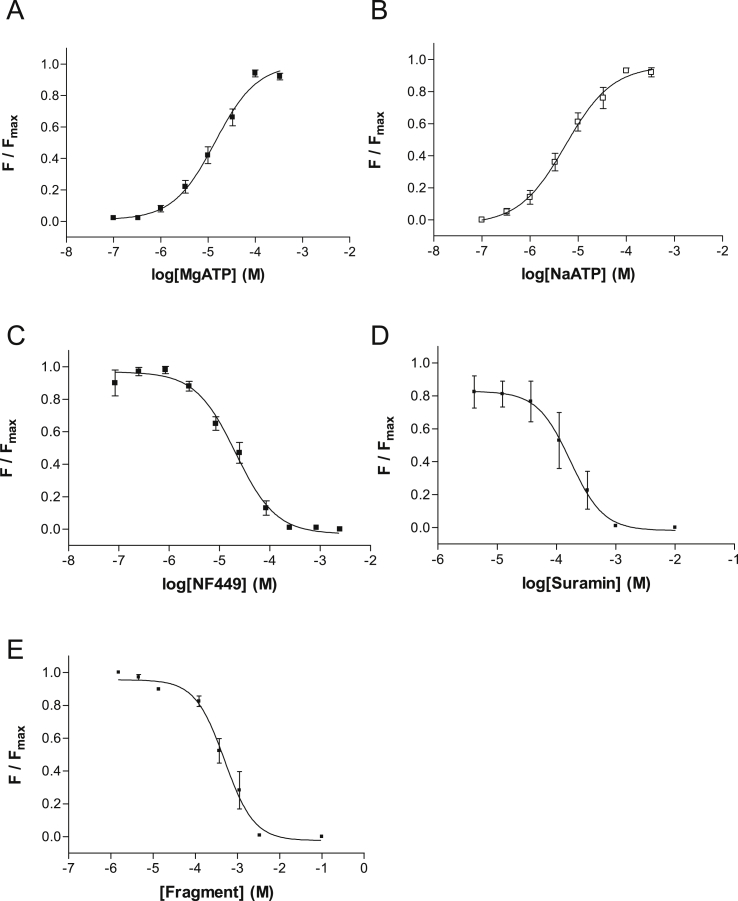
Properties of stably expressed doxycycline-inducible P2X1 receptors in a HEK 293T cell line. (A–B) Concentration–response curves for NaATP and MgATP, measured using a voltage-sensitive dye. (C–D) Concentration–inhibition of 10 μM ATP responses by known P2X1 antagonists NF449 and suramin. (E) Example data for one of the identified fragments.

Application of NaATP or MgATP to HEK 293T cells stably expressing the P2X1 receptor produced concentration-dependent fluorescent responses when loaded with a voltage-sensitive dye (Fig. 1, Fig. 2). Plotting peak fluorescent amplitude against a series of agonist concentrations allowed the data to be fitted with Eq. (1). For NaATP this gave a p*EC*_50_ of 5.26 ± 0.09 (*EC*_50_ = 5.49 μM, *n* = 22) and Hill slope of 0.9 ± 0.2, and for MgATP a p*EC*_50_ of 4.85 ± 0.07 (*EC*_50_ = 14.1 μM, *n* = 20) and Hill slope of 1.0 ± 0.1 (Fig. 2A and B). Untransfected HEK 293T cells did not respond to either NaATP or MgATP (Fig 1B). Agonist responses were completely inhibited by the known P2X1 receptor antagonists NF449 (p*IC*_50_ = 4.68 ± 0.06, *IC*_50_ = 20.1 μM, Hill Slope = 1.1 ± 0.2, *n* = 7; Fig. 2C) and suramin (p*IC*_50_ = 3.76 ± 0.15, *IC*_50_ = 174 μM, Hill Slope = 1.5 ± 0.7, *n* = 7; Fig. 2D).

### 3.2 Fragment screening

1443 fragments were screened at least twice and scored as hits when activity was observed on two plates. Using a final concentration of 300 μM, a total of 46 active fragments were identified as hits, which is an overall hit rate of 3.2% that compares well with other FBDD screens (Albert et al., 2007, Siegal et al., 2007, Chen and Hubbard, 2009, Davies et al., 2009, Gupta et al., 2009). At the end of this first screen the single point measurements were used to forecast the *EC*_50_ and *IC*_50_ values of the fragments using Eq. (1) and assuming a Hill slope of 1 (Fig 1C).

For identified hits, a second screen was used to verify concentration-dependence and to compare the resultant *EC*_50_ and *IC*_50_ values with those predicted from the hit screens. Concentration-dependence was examined using the same design as the hit screens, except a range of concentrations were used rather than a single concentration. The cells were similarly subjected to a second application of 30 μM ATP (10 μM final) to assess both agonist and antagonist behaviours. These results show that for the majority (39/46), concentration-dependence was confirmed, representing an 85% success rate for identifying hits in the initial hit screens and subsequently measuring concentration-dependence. When these *EC*_50_ and *IC*_50_ values were compared to estimates from the single point measurements they were generally higher (Fig 1C).

### 3.3 Properties of Alexa-647-ATP

**Fig. 3:**
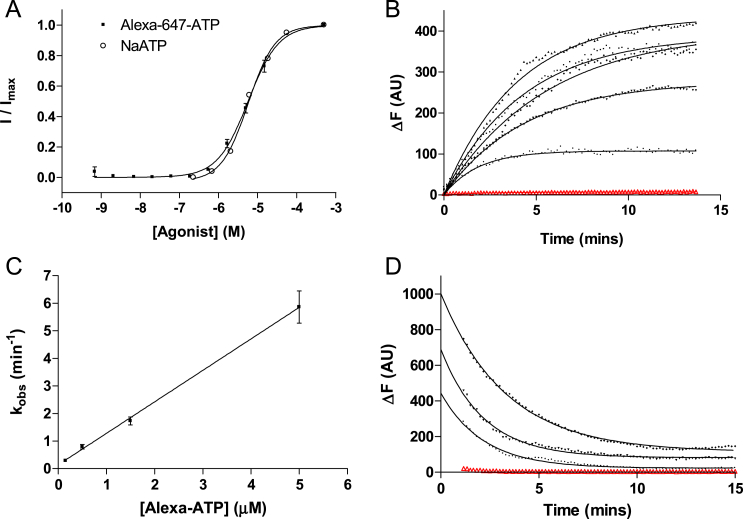
Kinetics of Alexa-647-ATP interactions at P2X1 receptors. (A) Alexa-647-ATP shows the same concentration-dependence as NaATP when measured using the voltage-sensitive dye. (B) Using confocal microscopy, the association of Alexa-647-ATP was determined by measuring the increase in cell-surface fluorescence following the application of different concentrations of this ligand. Upon application of Alexa-647-ATP a saturable increase in fluorescence was observed and was best fitted with a mono-exponential curve to yield *k*_obs_ (see Table 1). In this example the change in fluorescence following the addition of 0.3 μM Alexa-647-ATP is shown for both the background (*red*) and for five cells (*black*). (C) A plot of the average *k*_obs_ against the concentration of Alexa-647-ATP was fitted by linear regression (R^2^ = 0.89) to give the rates of association (slope) and dissociation (intercept at *y* = 0). The affinity of Alexa-647-ATP was calculated using these kinetic values (Eq. (3)) and gave a *K*_d_ of 119 nM. (D) Dissociation was measured after rapidly washing Alexa-647-ATP equilibrated P2X1-expressing HEK 293T cells with fresh PBS (*t* = 0). Dissociation was best-fitted with mono-exponential fits that gave an average dissociation rate (*k*_off_ = 0.40 min^−1^) that was similar to the value calculated in panel (C). Similar to panel A, there is low background fluorescence (red) when compared to cell-surface fluorescence (black).

To further validate the results of fragment screening another fluorescence method was used. Here we observed the competition between our hit fragments and the fluorophore Alexa-647-ATP, using confocal microscopy (Fig 3). As the properties of Alexa-647-ATP at P2X1 receptors are largely unknown it was first necessary to make measurements of Alexa-647-ATP alone.

Application of Alexa-647-ATP to P2X1 expressing cells produced a concentration-dependent fluorescent response when loaded with a voltage-sensitive dye (Fig 3A). Plotting peak fluorescent amplitude against a series of concentrations gave a p*EC*_50_ of 5.24 ± 0.04 (*EC*_50_ = 5.75 μM, *n* = 5) and Hill slope of 1.1 ± 0.1, that was similar to the values for NaATP and MgATP.

To characterise Alexa-647-ATP in more detail, confocal microscopy was used to make kinetic measurements. Following the application of Alexa-647-ATP to P2X1-expressing HEK 293T cells, the subsequent increase in fluorescence was well fitted by mono-exponential functions (Fig. 3B, Table 1). When the reciprocal of the rates of increase were plotted against Alexa-647-ATP concentration, the observed rate of association (τ_on_) increased linearly with the ligand concentration, as predicted by Eq. (2) (Fig. 3C). The association rate (*k*_on_) was determined from the slope of the 1/τ_on_ curve and the dissociation rate constant from the *y*-axis intercept. The association rate for Alexa-647-ATP was 1.142 × 10^6^ M^−1^ s^−1^, and the dissociation rate was 0.136 s^−1^, giving a *K*_b_ of 119 nM (Eq. (3)); direct measurements of dissociation gave a similar value for *k*_off_ (0.407 min^−1^, Fig 3D) and, consistent with a simple bi-molecular reaction scheme, the dissociation rate of Alexa-647-ATP was independent of the initial Alexa-647-ATP concentration (*data not shown*).

### 3.4 Confocal assessment of identified hits

**Fig. 4:**
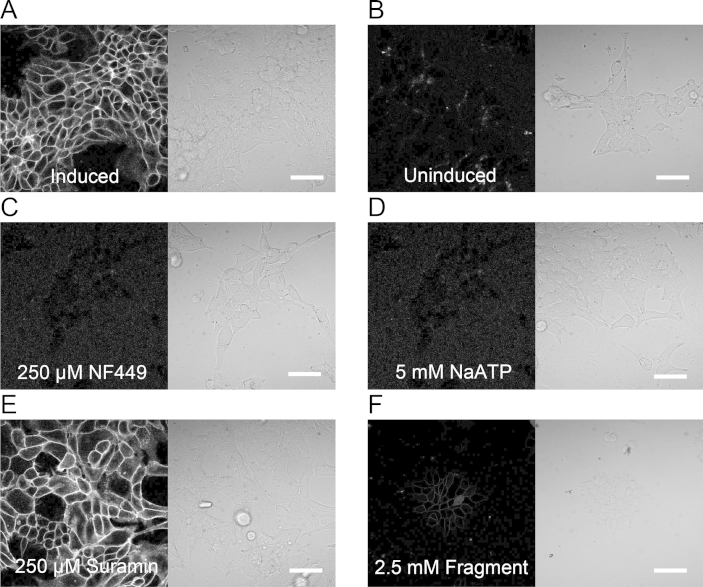
Confocal imaging of fluorescently-labelled P2X1 receptors expressed on the cell-surface of stably expressing HEK 293T cells. (A) Following an application of 150 nM Alexa-647-ATP a clear halo of fluorescence was visible on the cell-surface. Despite there being no washing steps in the preparation, the level of background fluorescence is negligible. (B) No fluorescence was seen on uninduced HEK 293T cells. (C, D) Fluorescent-labelling was not seen at P2X1 receptor-expressing HEK 293T cells that were pre-incubated with 250 μM NF449 or 5 mM non-labelled ATP. (E) Suramin did not alter cell-surface fluorescence. (F) Pre-incubation with 43/46 hits affected cell-surface labelling with Alexa-647-ATP. Panel F shows an example of one of these. All panels in this figure were measured using identical conditions. Scale = 50 μm.

**Fig. 5:**
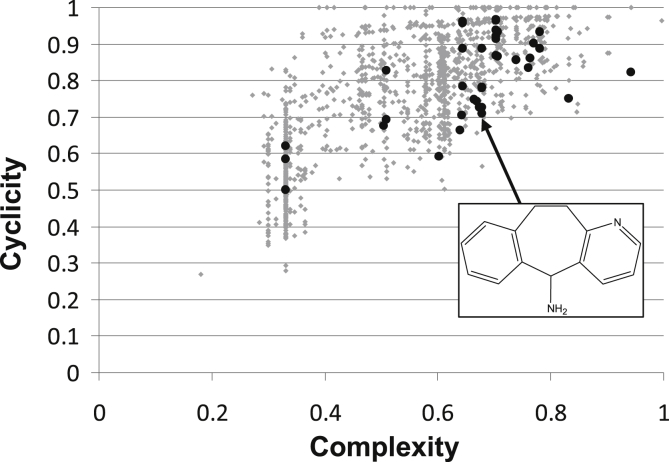
A scaffold-based classification approach (SCA) illustrates the high structural diversity of the identified hits. Cyclicity is a measure for the distribution of cyclic and acyclic parts of the structure, and complexity is a measure for size and shape of the scaffold (Xu, 2002).

Addition of 150 nM Alexa-647-ATP to P2X1 receptor-expressing HEK 293T cells resulted in a rapid increase in cell-surface fluorescence with very low background levels (Fig 4A). This fluorescence was not present on the surface of non-induced cells (Fig 4B) or on the surface of cells pre-treated with 250 μM NF449 (Fig 4C) or 5 mM NaATP (Fig 4D). Cell-surface fluorescence was still seen following pre-treatment with suramin (Fig 4E). Fluorescence was not seen inside labelled cells, indicating that Alexa-647-ATP was not cell-permeable.

Of the identified ligands from hit screens, 94% (43/46) also prevented binding of Alexa-647-ATP (Fig 4F). These results suggest that the majority of identified hit fragments compete with Alexa-647-ATP and further confirm our identification of hits. Negative hits were similarly assessed and did not compete with Alexa-647-ATP.

### 3.5 Structural diversity of the identified hit fragments

A high structural diversity of identified hits is illustrated by the Scaffold Classification Approach (SCA) as developed by Xu (2002). The plot in Fig 5 shows the high structural diversity of both agonists and antagonists as hits can be found throughout the scaffold space.

### 3.6 Discussion

To accurately characterise the pharmacology of a receptor it is necessary to have suitable ligands and experimental methods. Here we describe a fluorometric microplate assay for identifying candidate P2X1 receptor ligands using fragment-based drug discovery. This fluorescence-based assay provides a flexible and cost-effective solution for identifying new leads from compound libraries, and uses a voltage-sensitive dye that also makes it widely relevant for screening other ligand-gated ion channels. The assay also has several advantages over some traditional approaches such as radioligand binding and electrophysiology as it does not focus on a single binding site (unlike competition-based methods), it is suitable for extended compound pre-incubations, it is able to identify both agonists and antagonists in a single screen, and it overcomes some of the challenges associated with the rapid desensitisation of the P2X1 receptor.

46 hits were identified from a total of 1443 fragments and the activity of the majority of these confirmed by demonstrating concentration-dependence and observing competition with Alexa-647-ATP. In our initial hit-screens relatively high concentrations of fragments were used (300 μM), which is typical of fragment screening where higher starting concentrations are often needed and potency is later improved by chemical elaborations (Scott et al., 2012). Further experiments to determine their *EC*_50_ and *IC*_50_ values indicated that the majority of these candidate ligands (74%) had micro-molar affinities. Comparable levels of potency have been reported elsewhere when using a fragment-based approach, including targets as diverse as β2-adrinergic receptors, histamine receptors, protein kinase C, cAMP-specific 3′,5'-cyclic phosphodiesterase 4D and acetylcholine binding protein, β-secretase and BACE-1 (Murray et al., 2007, Wang et al., 2010, Scott et al., 2012, de Graaf et al., 2013). Importantly, when we have used a similar fluorometric approach on other ligand-gated ion channels we also identified novel fragments, showing the general applicability of the approach (Thompson et al., 2012, Verheij et al., 2012, Thompson et al., 2013).

Different analytical methods often give varying potencies for the same ligand and for NaATP and MgATP our *EC*_50_ values were elevated when compared to previously published work (Rettinger and Schmalzing, 2004). The standards suramin and NF449 also had *IC*_50_ values that were higher than those reported elsewhere; although literature values vary from μM – nM, depending upon the experimental system (Braun et al., 2001, El-Ajouz et al., 2012). However, these differences have been seen with voltage-sensitive dyes in other studies and may result from the use of a secondary reporter (the dye) to reveal currents in cells that are not voltage-clamped. Furthermore, if the apparent *EC*_50_ of the agonist is elevated, higher concentrations of antagonists may be needed to overcome the dextral shift of the inhibition curves caused by competition. Despite these differences, the assay was able to assay know ligands and identify ligands, while concentration-dependence provided a validation of the single point screens and indicated that the method provides a suitable screen for early hit identification that can be followed later by higher-resolution approaches on lower numbers of selected compounds (Kennedy et al., 2007, Jarvis and Khakh, 2009, El-Ajouz et al., 2012).

The *EC*_50_ for Alexa-647-ATP was close to the values for MgATP and NaATP, consistent with the comparable *EC*_50_ values of ATP and a fluorescent ATP derivative BODIPY-TR-ATP that have been reported at P2X3 receptors (Kowalski et al., 2014). These results suggest that these fluorescent agonists are suitable for studying ATP activation of P2X receptors using other fluorometric methods, such as in the voltage-clamp fluorometry reported by Bhargava et al. (2013) and in the single molecule microscopy by Barden et al. (2015). The finding that conjugation of a fluorophore does not affect the functional response is consistent with the crystal structures of ATP bound to P2X4 where it is clear that the ribose 2′ and 3′ positions onto which the dyes are substituted face out of the orthosteric binding site and would not be expected to overly affect ligand binding (Hattori and Gouaux, 2012). Other reports have shown low nanomolar *EC*_50_ values for both ATP and Alexa-647-ATP, but these are only apparent when desensitisation is dramatically slowed by substituting the N-terminus of P2X1 with that found in the more slowly desensitising P2X2 receptor (Rettinger and Schmalzing, 2004, Bhargava et al., 2013). Here, for the first time, a nanomolar affinity for Alexa-647-ATP was instead directly measured by determining *k*_off_ and *k*_on_ using confocal microscopy. Significantly, the affinity derived from these values (*K*_d_ = *k*_off_/*k*_on_) was similar to that measured using single molecule microscopy (Barden et al. 2015).

The affinity of Alexa-647-ATP was sufficiently high to enable live-cell imaging of expressed P2X1 receptors with very low background fluorescence. P2X1 and P2X3 receptors have a similar *EC*_50_ for ATP, and the latter has also been labelled with a fluorescent ATP derivative (Kowalski et al., 2014). In contrast, we could not detect any measureable fluorescence on the cell surface of HEK 293T cells expressing P2X4 or P2X7 receptors (*data not shown*), consistent with the lower potencies that these two receptor subtypes typically display for their ligands (Jarvis and Khakh, 2009). Of the 46 hits identified in our initial screens, 43 also reduced the Alexa-647-ATP fluorescent signal in subsequent confocal assays, a 94% success rate that is slightly higher than previous reports where two methods were used to validate compounds acting on ligand-gated ion channels (Thompson et al., 2010); in these studies hits from fluorescent microplate assays were validated by competition with a radiolabelled antagonist. For the remaining 3 fragments, 1 displayed neither concentration-dependence nor competition with Alexa-647-ATP suggesting that it was a false positive, while the other 2 fragments may be non-competitive or not potent enough to produce a measureable response in both assays. This may also be the reason that suramin caused functional inhibition in our fluorescent microplate assays, but no competition was seen under the confocal microscope, as both competitive and non-competitive modes of action have been described for this highly promiscuous ligand (Wong et al., 2000, Trujillo et al., 2006, Varani et al., 2008b). Previously, this combination of a functional assay and an assessment of orthosteric competition provided useful insights into the mechanisms of action of ligands and guided later medicinal chemistry efforts (Verheij et al., 2012, Thompson et al., 2013). An orthogonal approach has therefore become increasingly common, with the different methods often producing complimentary results.

To date there have been very few descriptions of P2X1 receptor ligands when compared to many other ligand-gated channels, but here we have shown that fragment-based drug discovery is a suitable approach for identify new leads (Gunosewoyo and Kassiou, 2010). Those that were identified belong to one of several structural clusters, as illustrated by the distribution of compounds in the SCA plot in Fig 5, and provide an ideal starting point for further medicinal chemistry. One of the clusters contains analogues of the tricyclic carbamazepines, and similar compounds have been reported elsewhere in the literature. Initially these were found to antagonise P2X4 receptors, but during the preparation of our manuscript have also been reported to bind to P2X1 receptors with micromolar affinities (Tian et al., 2014). Finding analogues of these compounds in our fragment library was reassuring and the potencies we measured for them were in the same micromolar range. Within other structural clusters there are several unique scaffolds which are now part of our on-going efforts to determine structure–activity relationships with the aim of optimising their potency and selectivity at P2X1 receptors.

The fluorescent methods presented here were developed to overcome the challenges of having limited quantities of test fragments and the need to rapidly screen large numbers of them on a ligand-gated ion channel. Elsewhere the Ca^2+^-sensitive dye Fluo-4 has been used to assess P2X4 ligands, supporting our notion that fluorescence-based methods provide a valuable research tool (Hernandez-Olmos et al., 2012). Other methods such as surface plasmon resonance (SPR), nuclear magnetic resonance (NMR), X-ray structure determination and mass spectrometry are also widely used for fragment screening, but are less commonly used on ligand-gated ion channels (de Kloe et al., 2009). Radioligand methods offer an alternative approach, but in our hands the high cost of a suitable P2X1 receptor radioligand made it impractical for routine screening (Varani et al., 2008a, Varani et al., 2008b). Electrophysiology could have been another possible alternative, but limits pre-application times and is complicated by rapid P2X1 receptor desensitisation that makes it difficult to measure the *IC*_50_ values of slowly equilibrating ligands (El-Ajouz et al., 2012, Bhargava et al., 2013). Methods such as these are therefore more practical for sensitive follow-up experiments on a smaller number of selected ligands and their derivatives, where complex phenomena such as modes of action and structure–activity relationships can be studied at higher resolution. In contrast, the fluorometric microplate method described here is a time- and cost-effective method that uses a voltage-sensitive dye that does not depend upon the availability of a high-affinity ligand, is ion-independent, can distinguish agonists from antagonists and, as the method is not limited to interactions at a single binding site, requires no presumptions regarding the site of action (Hulme and Trevethick, 2010). The assay is ideal for course screening to identify ligand candidates with actions at P2X1 receptors and whose properties can be subsequently improved by elaboration and further assessed using more time-consuming, higher-resolution methods.

## Authorship contributions

*Participated in research design*: AJT.

*Conducted experiments*: AJT.

*Contributed reagents or analytical tools*: M-DR, IdeE, RWF.

*Performed data analysis*: AJT, IdeE.

*Wrote or contributed to the writing of the manuscript*: AJT, IdeE, M-DR, RWF.

## Figures and Tables

**Table 1:** Rates of association of Alexa-647-ATP.

Alexa-647-ATP (μM)	*k*_obs_ (min^−1^)	*n*
0.15	0.28 ± 0.06	5
0.5	0.79 ± 0.08	7
1.5	1.73 ± 0.14	4
5	5.86 ± 0.58	6

## References

[bib1] Albert J.S., Blomberg N., Breeze A.L., Brown A.J., Burrows J.N., Edwards P.D. (2007). An integrated approach to fragment-based lead generation: philosophy, strategy and case studies from AstraZeneca's drug discovery programmes. Curr. Top. Med. Chem..

[bib2] Barden A.O., Goler A.S., Humphreys S.C., Tabatabaei S., Lochner M., Ruepp M.-D., Thompson A.J., Jones J.P., Brozik J.A. (2015). Tracking individual membrane proteins and their biochemistry: the power of direct observation. Neuropharmcol.

[bib3] Bhargava Y., Nicke A., Rettinger J. (2013). Validation of Alexa-647-ATP as a powerful tool to study P2X receptor ligand binding and desensitization. Biochem. Biophys. Res. Commun..

[bib4] Braun K., Rettinger J., Ganso M., Kassack M., Hildebrandt C., Ullmann H. (2001). NF449: a subnanomolar potency antagonist at recombinant rat P2X1 receptors. Naunyn Schmiedeb. Arch. Pharmacol..

[bib49] Congreve M., Carr R., Murray C., Jhoti H. (2003). A ‘rule of three’ for fragment-based lead discovery?. Drug Discov..

[bib5] Chen I.J., Hubbard R.E. (2009). Lessons for fragment library design: analysis of output from multiple screening campaigns. J. Comput. Aided Mol. Des..

[bib6] Darbousset R., Delierneux C., Mezouar S., Hego A., Lecut C., Guillaumat I. (2014). P2X1 expressed on polymorphonuclear neutrophils and platelets is required for thrombosis in mice. Blood.

[bib7] Davies D.R., Mamat B., Magnusson O.T., Christensen J., Haraldsson M.H., Mishra R. (2009). Discovery of leukotriene A4 hydrolase inhibitors using metabolomics biased fragment crystallography. J. Med. Chem..

[bib8] de Graaf C., Vischer H.F., de Kloe G.E., Kooistra A.J., Nijmeijer S., Kuijer M. (2013). Small and colorful stones make beautiful mosaics: fragment-based chemogenomics. Drug Discov. Today.

[bib9] de Kloe G.E., Bailey D., Leurs R., de Esch I.J. (2009). Transforming fragments into candidates: small becomes big in medicinal chemistry. Drug Discov. Today.

[bib10] El-Ajouz S., Ray D., Allsopp R.C., Evans R.J. (2012). Molecular basis of selective antagonism of the P2X1 receptor for ATP by NF449 and suramin: contribution of basic amino acids in the cysteine-rich loop. Br. J. Pharmacol..

[bib11] Fung C.Y., Cendana C., Farndale R.W., Mahaut-Smith M.P. (2007). Primary and secondary agonists can use P2X(1) receptors as a major pathway to increase intracellular Ca^2+^ in the human platelet. J. Thromb. Haemost..

[bib12] Fung C.Y., Jones S., Ntrakwah A., Naseem K.M., Farndale R.W., Mahaut-Smith M.P. (2012). Platelet Ca^2+^ responses coupled to glycoprotein VI and Toll-like receptors persist in the presence of endothelial-derived inhibitors: roles for secondary activation of P2X1 receptors and release from intracellular Ca^2+^ stores. Blood.

[bib13] Gever J.R., Cockayne D.A., Dillon M.P., Burnstock G., Ford A.P. (2006). Pharmacology of P2X channels. Pflugers Arch..

[bib14] Gunosewoyo H., Kassiou M. (2010). P2X purinergic receptor ligands: recently patented compounds. Expert Opin. Ther. Pat..

[bib15] Gupta A., Gupta A.K., Seshadri K. (2009). Structural models in the assessment of protein druggability based on HTS data. J. Comput. Aided Mol. Des..

[bib50] Hann M.M., Leach A.R., Harper G. (2001). Molecular complexity and its impact on the probability of finding leads for drug discovery. J Chem. Inf. Comput. Sci..

[bib16] Hattori M., Gouaux E. (2012). Molecular mechanism of ATP binding and ion channel activation in P2X receptors. Nature.

[bib17] Hernandez-Olmos V., Abdelrahman A., El-Tayeb A., Freudendahl D., Weinhausen S., Muller C.E. (2012). N-substituted phenoxazine and acridone derivatives: structure-activity relationships of potent P2X4 receptor antagonists. J. Med. Chem..

[bib18] Hulme E.C., Trevethick M.A. (2010). Ligand binding assays at equilibrium: validation and interpretation. Br. J. Pharmacol..

[bib19] Jackson S.P. (2007). The growing complexity of platelet aggregation. Blood.

[bib20] Jarvis M.F., Khakh B.S. (2009). ATP-gated P2X cation-channels. Neuropharmacology.

[bib21] Joseph-McCarthy D., Campbell A.J., Kern G., Moustakas D. (2014). Fragment-based lead discovery and design. J. Chem. Information Model..

[bib22] Kennedy C., Tasker P.N., Gallacher G., Westfall T.D. (2007). Identification of atropine- and P2X1 receptor antagonist-resistant, neurogenic contractions of the urinary bladder. J. Neurosci..

[bib23] Kowalski M., Hausmann R., Dopychai A., Grohmann M., Franke H., Nieber K. (2014). Conformational flexibility of the agonist binding jaw of the human P2X3 receptor is a prerequisite for channel opening. Br. J. Pharmacol..

[bib24] Macarron R. (2006). Critical review of the role of HTS in drug discovery. Drug Discov. Today.

[bib25] Marquis J., Kampfer S.S., Angehrn L., Schumperli D. (2009). Doxycycline-controlled splicing modulation by regulated antisense U7 snRNA expression cassettes. Gene Ther..

[bib26] Murray C.W., Callaghan O., Chessari G., Cleasby A., Congreve M., Frederickson M. (2007). Application of fragment screening by X-ray crystallography to beta-secretase. J. Med. Chem..

[bib27] Murray C.W., Verdonk M.L., Rees D.C. (2012). Experiences in fragment-based drug discovery. Trends Pharmacol. Sci..

[bib28] Price K.L., Lummis S.C. (2005). FlexStation examination of 5-HT(3) receptor function using Ca^2+^- and membrane potential-sensitive dyes: advantages and potential problems. J. Neurosci. Methods.

[bib51] Rees D.C., Congreve M., Murray C.W., Carr R. (2004). Fragment-based lead discovery. Nat. Rev. Drug Discov..

[bib29] Rettinger J., Schmalzing G. (2004). Desensitization masks nanomolar potency of ATP for the P2X1 receptor. J. Biol. Chem..

[bib30] Reymond J.-L., Ruddigkeit L., Blum L., van Deursen R. (2012). The enumeration of chemical space. WIREs Comput. Mol. Sci..

[bib31] Schuffenhauser A., Ruedisser S., Marzinzik A., Jahnke W., Blommers M., Selzer P. (2005). Library design for fragment based screening. Curr. Top. Med. Chem..

[bib32] Scott D.E., Coyne A.G., Hudson S.A., Abell C. (2012). Fragment-based approaches in drug discovery and chemical biology. Biochemistry.

[bib33] Siegal G., Ab E., Schultz J. (2007). Integration of fragment screening and library design. Drug Discov. Today.

[bib34] Thompson A.J., Verheij M.H., de Esch I.J., Lummis S.C. (2012). VUF10166, a novel compound with differing activities at 5-HT_3_A and 5-HT_3_AB receptors. J. Pharmacol. Exp. Ther..

[bib35] Thompson A.J., Verheij M.H., Leurs R., De Esch I.J., Lummis S.C. (2010). An efficient and information-rich biochemical method design for fragment library screening on ion channels. Biotechniques.

[bib36] Thompson A.J., Verheij M.H., van Muijlwijk-Koezen J.E., Lummis S.C., Leurs R., de Esch I.J. (2013). Structure-activity relationships of quinoxaline-based 5-HT_3_A and 5-HT_3_AB receptor-selective ligands. ChemMedChem.

[bib37] Tian M., Abdelrahman A., Weinhausen S., Hinz S., Weyer S., Dosa S. (2014). Carbamazepine derivatives with P2X4 receptor-blocking activity. Bioorg. Med. Chem..

[bib38] Tolhurst G., Carter R.N., Amisten S., Holdich J.P., Erlinge D., Mahaut-Smith M.P. (2008). Expression profiling and electrophysiological studies suggest a major role for Orai1 in the store-operated Ca^2+^ influx pathway of platelets and megakaryocytes. Platelets.

[bib39] Trujillo C.A., Nery A.A., Martins A.H., Majumder P., Gonzalez F.A., Ulrich H. (2006). Inhibition mechanism of the recombinant rat P2X2 receptor in glial cells by suramin and TNP-ATP. Biochemistry.

[bib40] Varani K., De Mattei M., Vincenzi F., Tosi A., Gessi S., Merighi S. (2008). Pharmacological characterization of P2X1 and P2X3 purinergic receptors in bovine chondrocytes. Osteoarthr. Cartilage/OARS, Osteoarthr. Res. Soc..

[bib41] Varani K., Surprenant A., Vincenzi F., Tosi A., Gessi S., Merighi S. (2008). Binding thermodynamic characterization of human P2X1 and P2X3 purinergic receptors. Biochem. Pharmacol..

[bib42] Varga-Szabo D., Braun A., Nieswandt B. (2009). Calcium signaling in platelets. J. Thromb. Haemost..

[bib43] Verheij M.H., Thompson A.J., van Muijlwijk-Koezen J.E., Lummis S.C., Leurs R., de Esch I.J. (2012). Design, synthesis, and structure-activity relationships of highly potent 5-HT_3_ receptor ligands. J. Med. Chem..

[bib44] Wang Y.S., Strickland C., Voigt J.H., Kennedy M.E., Beyer B.M., Senior M.M. (2010). Application of fragment-based NMR screening, X-ray crystallography, structure-based design, and focused chemical library design to identify novel microM leads for the development of nM BACE-1 (beta-site APP cleaving enzyme 1) inhibitors. J. Med. Chem..

[bib45] White C.W., Choong Y.T., Short J.L., Exintaris B., Malone D.T., Allen A.M. (2013). Male contraception via simultaneous knockout of α1A-adrenoceptors and P2X1-purinoceptors in mice. Proc. Natl. Acad. Sci. U. S. A..

[bib46] Wiznerowicz M., Trono D. (2003). Conditional suppression of cellular genes: lentivirus vector-mediated drug-inducible RNA interference. J. Virol..

[bib47] Wong A.Y., Burnstock G., Gibb A.J. (2000). Single channel properties of P2X ATP receptors in outside-out patches from rat hippocampal granule cells. J. Physiol..

[bib48] Xu J. (2002). A new approach to finding natural chemical structure classes. J. Med. Chem..

